# Trends in Prevalence of Overweight and Obesity in Korean Adults, 1998–2009: The Korean National Health and Nutrition Examination Survey

**DOI:** 10.2188/jea.JE20130017

**Published:** 2014-03-05

**Authors:** Hee-Taik Kang, Jae-Yong Shim, Hye-Ree Lee, Byoung-Jin Park, John A. Linton, Yong-Jae Lee

**Affiliations:** 1 Department of Family Medicine, Yonsei University College of Medicine, Seoul, Korea; 2 Department of Medicine, Graduate School, Yonsei University, Seoul, Korea; 3 Department of Family Medicine, College of Medicine, CHA University, Seoul, Korea; 4 International Health Care Center, Severance Hospital, Yonsei University College of Medicine, Seoul, Korea

**Keywords:** obesity, overweight, prevalence, trend

## Abstract

**Background:**

Although obesity is increasing worldwide and becoming a major public health problem, some countries report a trend toward stabilization. We investigated prevalence trends in overweight/obesity and obesity among Korean adults during a 12-year period.

**Methods:**

This study was based on the Korean National Health and Nutrition Examination Survey (KNHANES) I (1998), II (2001), III (2005), and IV (2007–2009). The χ^2^ and ANOVA tests were used to compare the prevalence and mean values for age and BMI, respectively. *P*-values for trends were determined by linear and logistic regression analyses, with KNHANES phase as the continuous variable.

**Results:**

The prevalences of overweight/obesity in KNHANES I through IV were 50.8%, 57.4%, 62.5%, and 62.6%, respectively, among men (*P* for trend = 0.002, β = 0.021) and 47.3%, 51.9%, 50.0%, and 48.9% among women (*P* for trend = 0.017, β = −0.015). The respective prevalences of obesity were 26.0%, 32.4%, 35.1%, and 36.3% among men (*P* for trend = 0.006, β = 0.018) and 26.5%, 29.3%, 28.0%, and 27.6% among women (*P* for trend = 0.143, β = −0.008). During the same period, the respective prevalences of grade 2 obesity (BMI ≥30 kg/m^2^) were 1.7%, 2.8%, 3.6%, and 3.8% among men (*P* for trend = 0.075, β = 0.005) and 3.0%, 3.5%, 3.4%, and 4.0% among women (*P* for trend = 0.398, β = 0.003).

**Conclusions:**

The prevalences of overweight/obesity and obesity showed an upward trend among men during the 12-year period, whereas the prevalence of overweight/obesity slightly decreased among women from 2001.

## INTRODUCTION

Obesity is a global epidemic.^1^^,^^2^ It is associated with numerous disorders, such as type 2 diabetes mellitus, metabolic syndrome, renal impairment, cancers, and cardiovascular diseases, which results in increased medical expenditures and substantial public health burdens.^3^^–^^6^ Although several studies have predicted that obesity prevalence will increase sharply,^1^^,^^2^ recent epidemiologic findings suggest that those reports overestimated future obesity prevalence.^7^^,^^8^

Rapid socioeconomic development and industrialization in South Korea over the last several decades have resulted in considerable lifestyle changes, such as increased consumption of Western food and sedentary behavior with less physical activity. Obesity and its related diseases, such as metabolic syndrome, have increased with the introduction of the Western lifestyle in Korea.^9^ Despite the gradual increase in obesity prevalence over several decades, the rate of increase is expected to slow or even reverse to a decrease in the next several years.^7^^,^^8^^,^^10^ Indeed, recent Japanese and Taiwanese epidemiologic studies have shown that female adiposity has decreased.^11^^,^^12^ Identifying changes in trends in obesity prevalence during recent decades would be helpful in establishing public-health promotion policies.

Using data from the Korean National Health and Nutrition Examination Survey (KNHANES) for the period 1998–2009, we investigated prevalence trends in overweight and obesity among Korean adults aged 20 years or older.

## METHODS

### Study population

The KNHANES is a cross-sectional, nationally representative survey conducted by the Korean Ministry of Health and Welfare. It assesses the health and nutritional status of the non-institutionalized Korean population and has been performed in 4 phases: phase I (1998), II (2001), III (2005), and IV (2007–2009). KNHANES comprises a health interview survey, a health behavior survey, a health examination survey, and a nutrition survey. Households as sampling units were stratified and collected through a multistage, probability-based sampling design based on sex, age, and geographic area, using household registries. At the time each survey was done, participants provided written informed consent for use of their data in further analyses and were given the right to refuse to participate, in accordance with the National Health Enhancement Act.

Of the 138 674 participants in the KNHANES I to IV, those who were 19 years or younger (*n* = 40 821) or had missing anthropometric data (*n* = 60 731) were excluded. After these exclusions, a total of 37 122 participants (16 040 men, 21 082 women) were included in the final analysis. Age group at the time of the interview was classified as young adults (20–39 years), middle-aged adults (40–59 years), and elderly adults (60 years or older). This study was approved by the Institutional Review Board of Gangnam Severance Hospital, Yonsei University College of Medicine, Seoul, Korea.

### Measurement and definition of obesity

Physical examinations were performed by trained medical staff who followed standardized procedures. Body weight and height were measured to the nearest 0.1 kg and 0.1 cm, respectively, with subjects wearing light indoor clothing without shoes. Body mass index (BMI) was calculated as the ratio of weight in kilograms to height in meters squared (kg/m^2^). Using the Asia-Pacific regional guidelines of the World Health Organization (WHO) and International Obesity Task Force (IOTF) we defined the cutoff points for overweight and obesity as a BMI of 23 kg/m^2^ or higher and 25 kg/m^2^ or higher, respectively.^13^ We further stratified obesity as grade 1 (BMI ≥25 kg/m^2^) and grade 2 (BMI ≥30 kg/m^2^), which were not mutually exclusive.

### Statistical analysis

KNHANES data obtained from the Korea National Statistical Office were used to define the standard population. To represent the whole Korean population without biased estimates, sampling weights were used to account for the complex sampling. The age and BMI of the study population were summarized using general linear models across KNHANES phases. The χ^2^ test was used to compare obesity prevalence, and 1-way analysis of variance (ANOVA) was used to compare mean values for continuous variables such as age and BMI, across KNHANES phases. All data on continuous variables are presented as mean ± standard error (SE). Data on categorical variables are presented as percentage and 95% CI. Odds ratios (ORs) and 95% CI for the obesity prevalence were calculated using logistic regression analyses across KNHANES phases by sex, after adjusting for age group. When we conducted trend analyses, KNHANES phases I to IV were expressed by numerical values, which were treated as continuous rather than categorical variables. We conducted linear regression analyses for BMI and logistic regression analyses for prevalence and ORs, to calculate *P*-values for trends and β coefficients across KNHANES phases. All analyses were conducted using SAS statistical software (version 9.1; SAS Institute Inc., Cary, NC, USA). All statistical tests were 2-sided, and a *P*-value of less than 0.05 was considered to indicate statistical significance.

## RESULTS

The 37 122 participants included in this study were distributed as follows: 7962 from KNHANES I, 6572 from KNHANES II, 5462 from KNHANES III, and 17 126 from KNHANES IV (Table 1).

**Table 1.** Number (percentage) of study subjects according to KNHANES phase, age group, and sex

	I	II	III	IV	Total
Men, *N*	3597 (100)	2864 (100)	2325 (100)	7254 (100)	16 040 (100)
20–39 years	1582 (44.0)	1192 (41.6)	768 (33.0)	2354 (32.5)	5896 (36.8)
40–59 years	1350 (37.5)	1125 (39.3)	1003 (43.2)	2720 (37.5)	6198 (38.6)
≥60 years	665 (18.5)	547 (19.1)	554 (23.8)	2180 (30.0)	3946 (24.6)

Women, *N*	4365 (100)	3708 (100)	3137 (100)	9872 (100)	21 082 (100)
20–39 years	1879 (43.0)	1561 (42.1)	1128 (35.9)	3230 (32.7)	7798 (37.0)
40–59 years	1540 (35.3)	1343 (36.2)	1248 (39.8)	3578 (36.3)	7709 (36.6)
≥60 years	946 (21.7)	804 (21.7)	761 (24.3)	3064 (31.0)	5575 (26.4)

Table 2 shows changes in BMI according to sex and age group (20–39 years, 40–59 years, 60 years or older) across KNHANES phases. BMI increased among men across KNHANES phases (*P*-value ≤0.001 by 1-way ANOVA; *P* for trend <0.001, β = 0.169 by linear regression analysis); however, BMI slightly decreased among women (*P*-value = 0.013; *P* for trend = 0.035, β = −0.093).

**Table 2.** Weighted estimates of body mass index (BMI), based on KNHANES (1998–2009)

Sex		I	II	III	IV	*P*-value	*P* for trend	β coefficient
Men	Age (years)	42.2 ± 0.32	45.7 ± 0.38	42.8 ± 0.44	43.7 ± 0.27	<0.001		
	BMI (kg/m^2^)							
	All	23.2 ± 0.07	23.7 ± 0.07	24.0 ± 0.08	24.0 ± 0.05	<0.001	<0.001	0.169
	20–39 years	23.1 ± 0.10	23.6 ± 0.11	23.7 ± 0.14	24.1 ± 0.08	<0.001	0.001	0.237
	40–59 years	23.7 ± 0.09	24.1 ± 0.09	24.4 ± 0.11	24.3 ± 0.07	<0.001	0.210	0.071
	≥60 years	22.0 ± 0.13	23.0 ± 0.17	23.4 ± 0.18	23.3 ± 0.10	<0.001	0.115	0.149

Women	Age (years)	43.3 ± 0.39	45.6 ± 0.39	44.6 ± 0.41	45.8 ± 0.28	<0.001		
	BMI (kg/m^2^)							
	All	23.1 ± 0.06	23.4 ± 0.07	23.3 ± 0.09	23.2 ± 0.05	0.013	0.035	−0.093
	20–39 years	22.2 ± 0.08	22.2 ± 0.09	22.2 ± 0.12	22.0 ± 0.08	0.396	0.139	−0.092
	40–59 years	24.1 ± 0.09	24.2 ± 0.10	24.2 ± 0.12	23.9 ± 0.07	0.017	0.002	−0.184
	≥60 years	23.8 ± 0.13	24.3 ± 0.16	24.3 ± 0.14	24.4 ± 0.08	0.002	0.708	0.033

All data are mean ± SE; *P*-values were determined by 1-way ANOVA.

*P*-values for trends and β coefficient were determined by linear regression analyses after setting KNHANES phase as the continuous variable.

**Figure 1.**
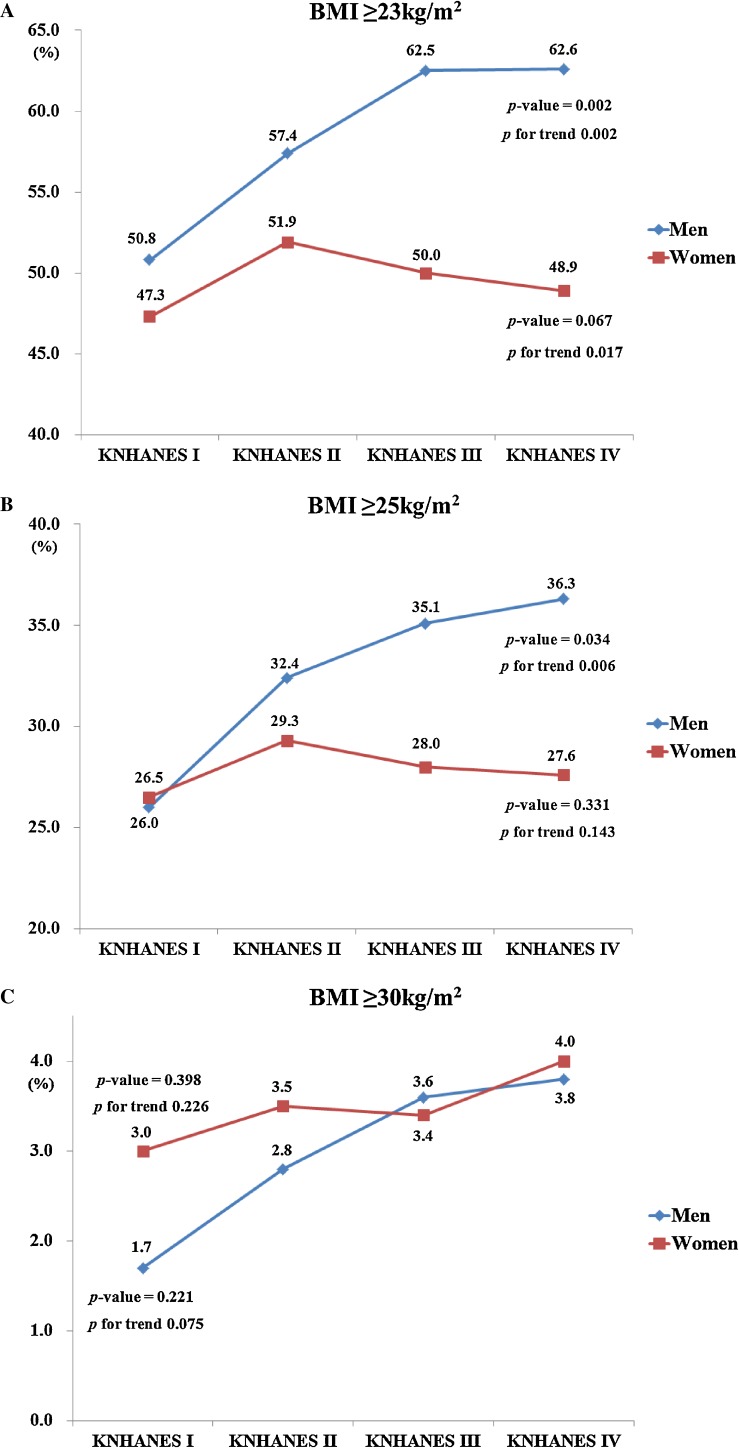
Prevalence of overweight/obesity, grade 1 obesity, and grade 2 obesity in men and women, according to KNHANES phase. *P*-values represent difference in prevalences of overweight/obesity, grade 1 obesity, and grade 2 obesity among KNHANES phases (calculated by χ^2^ test). *P*-values for trends were determined by logistic regression analyses after setting KNHANES phase as the continuous variable. Definition: overweight/obesity, BMI ≥23 kg/m^2^; grade 1 obesity, BMI ≥25 kg/m^2^; grade 2 obesity, BMI ≥30 kg/m^2^.

The prevalences of overweight/obesity (BMI ≥23 kg/m^2^), grade 1 obesity (BMI ≥25 kg/m^2^), and grade 2 obesity (BMI ≥30 kg/m^2^) for men and women are presented in Figure 1. The prevalence of overweight/obesity gradually rose among men from KNHANES I to IV (*P*-value = 0.002 by χ^2^ test; *P* for trend = 0.002, β = 0.021 by logistic regression analysis) but decreased among women (*P*-value = 0.067; *P* for trend = 0.017, β = −0.015; Figure 1A). Similar to the trends in overweight/obesity prevalence, the overall prevalence of grade 1 obesity steadily increased among men (*P*-value = 0.034; *P* for trend = 0.006, β = 0.018), but the trend was not significant among women, although it tended to decrease after KNHANES II (*P*-value = 0.331; *P* for trend = 0.143, β = −0.008; Figure 1B). In contrast, the increase in the prevalence of grade 2 obesity was not significant in either sex (*P*-value = 0.221 in men and 0.398 in women; *P* for trend = 0.075 in men and 0.226 in women, β = 0.005 in men and 0.003 in women; Figure 1C).

Detailed information on the prevalences of overweight/obesity, grade 1 obesity, and grade 2 obesity according to age group and sex are shown in Table 3. The prevalence of overweight/obesity (*P*-value = 0.037 by χ^2^ test; *P* for trend = 0.006 and β = 0.030 by logistic regression analysis) and grade 2 obesity (*P*-value = 0.165; *P* for trend = 0.041, β = 0.010) gradually increased in young adult men across KNHANES phases. In contrast, the prevalence of overweight/obesity (*P*-value = 0.048; *P* for trend = 0.012, β = −0.022) among young adult women and the prevalences of overweight/obesity (*P*-value = 0.028; *P* for trend = 0.008, β = −0.025) and grade 1 obesity (*P*-value = 0.042; *P* for trend = 0.010, β = −0.023) among middle-aged women linearly decreased across KNHANES phases.

**Table 3.** Prevalence of overweight/obesity, grade 1 obesity (BMI ≥25 kg/m^2^), and grade 2 obesity (≥30 kg/m^2^), based on KNHANES (1998–2009)

Men	I	II	III	IV	*P*-value	*P* for trend	β coefficient
BMI ≥23 kg/m^2^							
20–39 years	48.0 (44.9–51.2)	54.5 (51.3–57.6)	58.2 (54.2–62.1)	60.7 (58.2–63.1)	0.037	0.006	0.030
40–59 years	60.4 (57.4–63.2)	65.1 (61.8–68.2)	70.0 (66.5–73.3)	67.8 (65.6–70.0)	0.156	0.475	0.007
≥60 years	35.1 (30.9–39.5)	48.6 (43.2–54.2)	57.0 (51.3–62.5)	54.9 (52.0–57.9)	0.074	0.104	0.025

BMI ≥25 kg/m^2^							
20–39 years	24.7 (22.4–27.2)	32.0 (28.7–35.4)	32.0 (28.5–35.7)	35.4 (33.1–37.6)	0.130	0.052	0.020
40–59 years	31.3 (28.2–34.5)	36.3 (33.1–39.6)	41.1 (37.4–44.8)	40.8 (38.7–43.0)	0.105	0.069	0.018
≥60 years	16.3 (13.4–19.8)	26.2 (21.6–31.4)	29.7 (24.8–35.1)	27.6 (25.1–30.2)	0.619	0.799	0.003

BMI ≥30 kg/m^2^							
20–39 years	2.2 (1.5–3.2)	3.5 (2.4–5.0)	4.8 (3.2–7.0)	5.6 (4.6–6.8)	0.165	0.041	0.010
40–59 years	1.6 (1.1–2.4)	2.5 (1.7–3.7)	3.2 (2.0–5.0)	2.8 (2.2–3.6)	0.713	0.921	0.000
≥60 years	0.1 (0.0–0.7)	2.1 (0.9–4.8)	1.2 (0.6–2.5)	1.6 (1.0–2.7)	0.647	0.782	−0.001



Women	I	II	III	IV	*P*-value	*P* for trend	β coefficient
BMI ≥23 kg/m^2^							
20–39 years	33.6 (31.1–36.2)	35.5 (32.8–38.2)	32.7 (29.6–35.9)	31.0 (29.0–33.1)	0.048	0.012	−0.022
40–59 years	60.3 (57.4–63.3)	62.6 (59.3–65.8)	61.6 (58.0–65.2)	57.9 (55.9–59.9)	0.028	0.008	−0.025
≥60 years	59.6 (56.0–63.2)	63.5 (59.1–67.6)	66.6 (62.5–70.6)	66.4 (63.9–68.8)	0.441	0.300	0.013

BMI ≥25 kg/m^2^							
20–39 years	17.3 (15.5–19.3)	16.5 (14.6–18.5)	16.6 (14.1–19.4)	15.9 (14.4–17.5)	0.846	0.601	−0.003
40–59 years	35.3 (32.4–38.3)	36.3 (33.2–39.4)	34.5 (31.2–38.0)	31.8 (30.0–33.7)	0.042	0.010	−0.023
≥60 years	34.9 (31.4–38.5)	41.0 (37.0–45.2)	41.0 (37.1–45.0)	42.0 (39.6–44.5)	0.866	0.640	0.006

BMI ≥30 kg/m^2^							
20–39 years	2.2 (1.6–3.1)	1.9 (1.3–2.8)	2.7 (1.9–3.9)	3.0 (2.3–3.8)	0.184	0.071	0.005
40–59 years	3.5 (2.7–4.6)	3.9 (2.8–5.3)	4.7 (3.4–6.4)	4.3 (3.5–5.3)	0.749	0.679	0.002
≥60 years	4.2 (3.1–5.7)	5.6 (3.9–8.1)	2.8 (1.8–4.2)	5.1 (4.0–6.3)	0.051	0.959	0.000

*P*-values were determined by χ^2^ testing.

*P*-values for trends and β coefficient were determined by logistic regression analyses after setting KNHANES phase as the continuous variable.

**Figure 2.**
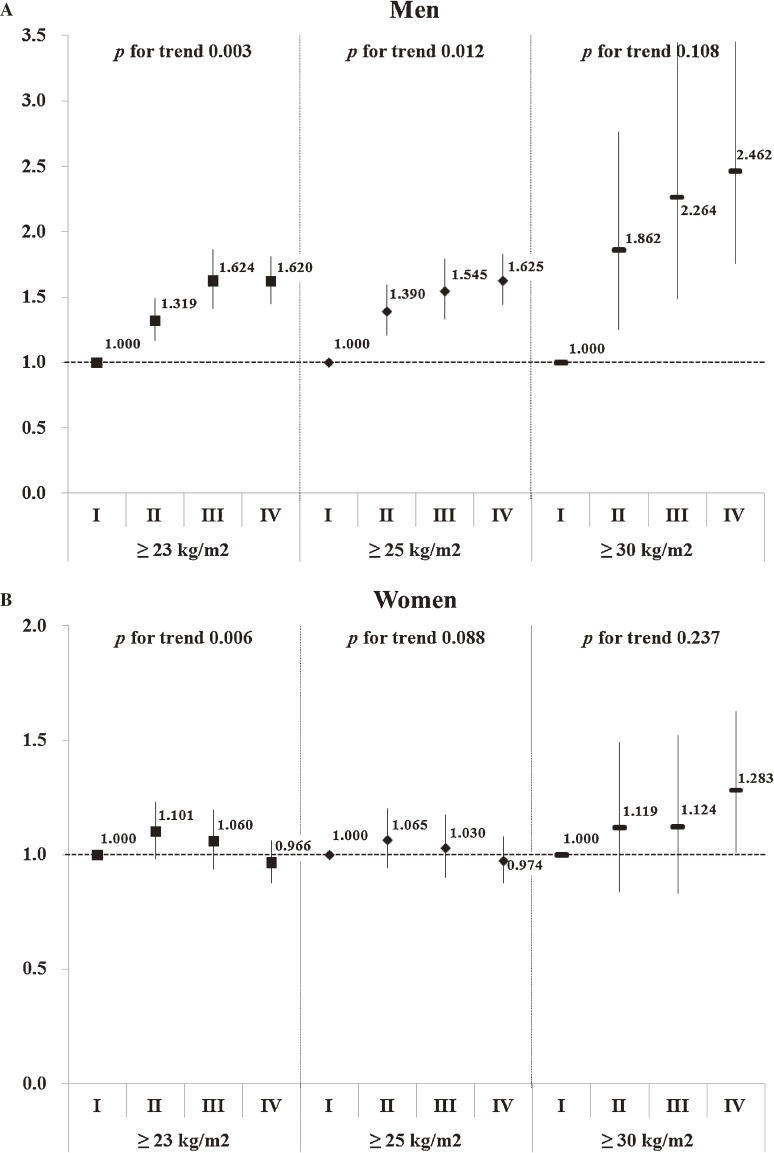
Odds ratios estimated by adjusting age groups (20–39 years, 40–59 years, and ≥60 years) for the prevalence of overweight and obesity in men and women, according to KNHANES phase. *P*-values for trends were determined by logistic regression analyses after setting KNHANES phase as the continuous variable. Definition: overweight/obesity, BMI ≥23 kg/m^2^; grade 1 obesity, BMI ≥25 kg/m^2^; grade 2 obesity, BMI ≥30 kg/m^2^.

The trends in overweight and obesity during 1998–2009 are presented as ORs (95% CIs) indicating the estimated increase in the odds for obesity prevalence after adjusting for age group (Figure 2). As compared with KNHANES I, the ORs in men for overweight/obesity in KNHANES II to IV were all statistically significant and linearly increased in a time-dependent manner (1.319 [1.166–1.492] in KNHANES II, 1.624 [1.413–1.865] in KNHANES III, and 1.620 [1.448–1.812] in KNHANES IV, *P* for trend = 0.003; Figure 2A), while those in women were all insignificant (1.101 [0.983–1.232] in KNHANES II, 1.060 [0.938–1.198] in KNHANES III, and 0.966 [0.877–1.063] in KNHANES IV; Figure 2B). The ORs for grade 1 obesity among men in KNHANES II through IV significantly increased and showed an upward trend (1.390 [1.209–1.598] in KNHANES II, 1.545 [1.333–1.792] in KNHANES III, and 1.625 [1.445–1.827] in KNHANES IV, *P* for trend = 0.012; Figure 2A). In contrast, those in women were not statistically significant (1.065 [0.944–1.202] in KNHANES II, 1.030 [0.902–1.177] in KNHANES III, and 0.974 [0.878–1.080] in KNHANES IV; Figure 2B). Furthermore, as compared with KNHANES I, ORs for grade 2 obesity were significantly increased in men across all KNHANES phases (1.862 [1.254–2.765] in KNHANES II, 2.264 [1.485–3.450] in KNHANES III, and 2.462 [1.754–3.455] in KNHANES IV; Figure 2A), while, in women, the OR was statistically significant only in KNHANES IV (1.283 [1.011–1.629]; Figure 2B).

## DISCUSSION

This study of KNHANES data for 1998–2009 showed sex differences in trends in overweight and obesity prevalence among Korean adults during the 12-year period. The trend in overweight and obesity prevalence increased linearly in men; however, there was no increasing trend for women, and overweight/obesity even decreased during the 12-year period. These findings are comparable to the results of other East Asian studies.^11^^,^^12^

BMI is a reasonable measure to assess general obesity.^14^ The WHO and IOTF recommend that overweight and obesity be defined as a BMI of 23 kg/m^2^ or higher and 25 kg/m^2^ or higher, respectively, in the Western Pacific region.^13^ High BMI is a well-known risk factor for cardiometabolic diseases such as type 2 diabetes mellitus, hypertension, and ischemic heart diseases.^3^^–^^6^ In addition, BMI independently predicts all-cause and cardiovascular mortality, especially in individuals with a BMI over 25 kg/m^2^.^15^^,^^16^

Direct and indirect medical expenditures and public health burdens related to obesity will increase as long as obesity prevalence increases.^1^ According to a systematic review, obesity was responsible for 0.7% to 2.8% of national total healthcare expenditures.^17^ Medical costs among obese individuals are about 30% higher than those of non-obese individuals.^17^ Unfortunately, obesity prevalence is markedly increasing, and this upward trend is likely to continue.^1^^,^^2^^,^^18^ A systematic analysis of health examination surveys from 199 countries and regions showed that mean BMI increased by 0.4 kg/m^2^ per decade in men and by 0.5 kg/m^2^ per decade in women between 1980 and 2008.^19^ However, the latest findings on changes in obesity prevalence in American children and adults contradict earlier results.^7^^,^^8^

In a recent study of Korean adults, which was based on data from KNHANES I–IV, Khang et al reported that obesity prevalence gradually increased in Korean men but plateaued in Korean women during 1998–2007.^10^ However, that study did not include data from 2008 and 2009, ie, the second and third years of the 3 years studied in KNHANES IV. For this reason, it was necessary to perform further trend analyses to observe changes in obesity prevalence among Korean adults during a 12-year period, based on data from KNHANES I to IV.

Mean BMI continuously increased in men, from 23.2 kg/m^2^ in 1998 to 24.0 kg/m^2^ in 2009. The rate of BMI increase in Koreans exceeds the global average (0.67 kg/m^2^ vs 0.4 kg/m^2^ per decade). Female mean BMI increased slightly, from 23.1 kg/m^2^ in 1998 to 23.2 kg/m^2^ in 2009, but has decreased since 2001. The prevalence trend in overweight/obesity increased in men from 1998 to 2009 and has been decreasing in women since 2001. These findings are comparable to those of a Japanese study.^12^

An imbalance between daily calorie intake and expenditure is a major factor in body weight change. According to Korea Health Statistics, which was published by the Korea Centers for Disease Control and Prevention, Korean trends in daily calorie intake were as follows^20^: 2152 kcal, 2107 kcal, 2214 kcal, and 2148 kcal for men, and 1729 kcal, 1713 kcal, 1742 kcal, and 1579 kcal for women in KNHANES I to IV, respectively. Thus, a decrease in daily calorie intake of about 150 kcal was seen in women between 1998 and 2009, whereas a decrease of only 4 kcal was seen in men. Moreover, the percentage of individuals regularly engaged in physical activities of moderate or vigorous intensity changed from 33.3% to 27.3% in men and from 26.1% to 23.0% in women in the KNHANES III and IV, respectively. We infer that the small change in daily calorie intake and the large decrease in physical activity have made Korean men more obese, while the large decrease in daily calorie intake and the small decrease in physical activity have made Korean women less obese. Sex differences in the trends in changes in calorie intake and physical activity might result in contradictory trends in the prevalence of overweight and obesity. Several studies reported that more frequent exposure to mass media in a population increases body dissatisfaction and distorts eating patterns, especially in young women.^21^^,^^22^ Many Korean women exposed to mass media may wish to make their bodies more slender and might thus engage in excessive weight-reduction practices, regardless of their adiposity status. A desire to become more slender might have contributed to downward trends in overweight and obesity in women.

Some limitations should be considered when interpreting the findings of this study. First, this study analyzed cross-sectional data. In addition, many subjects (*n* = 60 731) were excluded because of missing anthropometric data. In particular, overweight or obese women might have avoided anthropometric measurements. For these reasons, we cannot fully exclude the possibility of selection bias, despite the enrollment of a large representative sample and use of sampling weights. Second, overweight and obesity were defined based on BMI, which is calculated using body weight and height and does not consider body fatness. Because BMI does not distinguish fat from fat-free mass and tends to overestimate fatness in muscular subjects, adiposity can be misclassified. However, BMI was found to be strongly correlated with fat-free mass and fat mass.^23^ Furthermore, BMI was well validated with body fat percentage as assessed by dual X-ray absorptiometry.^24^ For these reasons, it is unlikely that this method of assessing obesity significantly affected our findings. Finally, parameters of obesity, caloric intake, and physical activity were not measured over the same relevant phase. For instance, because the Korea Centers for Disease Control and Prevention did not comprehensively survey moderate- and vigorous-intensity physical activity before KNHANES III, we could not estimate the percentage of individuals who were regularly engaged in physical activity from KNHANES I to IV. These limitations make it difficult to identify conclusive cause–effect relationships.

Despite its potential limitations, this study has several strengths. First, to represent the general Korean population aged 20 years or older, we applied sampling weights to all analyses. A similar study, by Khang et al, enrolled a smaller population for a shorter period.^10^ Furthermore, they included only the first of 3 years of KNHANES IV. KNHANES began in 1998 (KNHANES I). By KNHANES III, this survey was conducted every 3 or 4 years for 2 to 3 months. Unlike the previous 3 surveys, KNHANES IV was conducted year-round from 2007 to 2009. Data from the first years of KNHANES IV, which Khang et al analyzed in their study, do not represent the general Korean population in the relevant period. Thus, our findings are more generalizable and representative of Korean adults.

In summary, the prevalence of overweight and obesity continuously increased in Korean men, and slightly decreased in women, during the 12-year study period 1998–2009. Appropriate national interventions, including public anti-obesity campaigns and health promotion programs, are warranted in order to reduce the prevalence of obesity.

## References

[1] Finkelstein EA, Khavjou OA, Thompson H, Trogdon JG, Pan L, Sherry B, Obesity and severe obesity forecasts through 2030. Am J Prev Med. 2012;42(6):563–70 10.1016/j.amepre.2011.10.026 22608371

[2] Wang Y, Beydoun MA, Liang L, Caballero B, Kumanyika SK Will all Americans become overweight or obese? estimating the progression and cost of the US obesity epidemic. Obesity (Silver Spring). 2008;16(10):2323–30 10.1038/oby.2008.351 18719634

[3] Logue J, Murray HM, Welsh P, Shepherd J, Packard C, Macfarlane P, Obesity is associated with fatal coronary heart disease independently of traditional risk factors and deprivation. Heart. 2011;97(7):564–8 10.1136/hrt.2010.211201 21324888

[4] Després JP, Lemieux I Abdominal obesity and metabolic syndrome. Nature. 2006;444(7121):881–7 10.1038/nature05488 17167477

[5] Park MH, Falconer C, Viner RM, Kinra S The impact of childhood obesity on morbidity and mortality in adulthood: a systematic review. Obes Rev. 2012;13:985–1000 10.1111/j.1467-789X.2012.01015.x 22731928

[6] Noori N, Hosseinpanah F, Nasiri AA, Azizi F Comparison of overall obesity and abdominal adiposity in predicting chronic kidney disease incidence among adults. J Ren Nutr. 2009;19(3):228–37 10.1053/j.jrn.2008.11.005 19261489

[7] Flegal KM, Carroll MD, Kit BK, Ogden CL Prevalence of obesity and trends in the distribution of body mass index among US adults, 1999–2010. JAMA. 2012;307(5):491–7 10.1001/jama.2012.39 22253363

[8] Ogden CL, Carroll MD, Kit BK, Flegal KM Prevalence of obesity and trends in body mass index among US children and adolescents, 1999–2010. JAMA. 2012;307(5):483–90 10.1001/jama.2012.40 22253364 PMC6362452

[9] Lim S, Shin H, Song JH, Kwak SH, Kang SM, Won Yoon J, Increasing prevalence of metabolic syndrome in Korea: the Korean National Health and Nutrition Examination Survey for 1998–2007. Diabetes Care. 2011;34(6):1323–8 10.2337/dc10-2109 21505206 PMC3114326

[10] Khang YH, Yun SC Trends in general and abdominal obesity among Korean adults: findings from 1998, 2001, 2005, and 2007 Korea National Health and Nutrition Examination Surveys. J Korean Med Sci. 2010;25(11):1582–8 10.3346/jkms.2010.25.11.1582 21060746 PMC2966994

[11] Chu NF Prevalence of obesity in Taiwan. Obes Rev. 2005;6(4):271–4 10.1111/j.1467-789X.2005.00175.x 16246212

[12] Yoshiike N, Seino F, Tajima S, Arai Y, Kawano M, Furuhata T, Twenty-year changes in the prevalence of overweight in Japanese adults: the National Nutrition Survey 1976–95. Obes Rev. 2002;3(3):183–90 10.1046/j.1467-789X.2002.00070.x 12164470

[13] International Obesity TaskForce & World Health Organization. The Asian-Pacific perspective: Redefining obesity and its treatment. 2000. Available from: http://www.wpro.who.int/nutrition/documents/Redefining_obesity/en/index.html (accessed January 31, 2013).

[14] Hu F. Obesity epidemiology. Oxford: Oxford University Press; 2008.

[15] Prospective Studies Collaboration, Whitlock G, Lewington S, Sherliker P, Clarke R, Emberson J, Halsey J, Body-mass index and cause-specific mortality in 900 000 adults: collaborative analyses of 57 prospective studies. Lancet. 2009;373(9669):1083–96 10.1016/S0140-6736(09)60318-4 19299006 PMC2662372

[16] Yun KE, Park HS, Song YM, Cho SI Increases in body mass index over a 7-year period and risk of cause-specific mortality in Korean men. Int J Epidemiol. 2010;39(2):520–8 10.1093/ije/dyp282 19762478

[17] Withrow D, Alter DA The economic burden of obesity worldwide: a systematic review of the direct costs of obesity. Obes Rev. 2011;12(2):131–41 10.1111/j.1467-789X.2009.00712.x 20122135

[18] Kuczmarski RJ, Flegal KM, Campbell SM, Johnson CL Increasing prevalence of overweight among US adults. The National Health and Nutrition Examination Surveys, 1960 to 1991. JAMA. 1994;272(3):205–11 10.1001/jama.1994.03520030047027 8022039

[19] Finucane MM, Stevens GA, Cowan MJ, Danaei G, Lin JK, Paciorek CJ, National, regional, and global trends in body-mass index since 1980: systematic analysis of health examination surveys and epidemiological studies with 960 country-years and 9.1 million participants. Lancet. 2011;377(9765):557–67 10.1016/S0140-6736(10)62037-5 21295846 PMC4472365

[20] Korea Centers for Disease Control and Prevention. Korea Health Statistics 2009: Korea National Health and Nutrition Examination Survey (KNHANES IV-3). 2012. Available from: http://knhanes.cdc.go.kr/ (accessed January 31, 2013).

[21] Moriarty CM, Harrison K Television Exposure and Disordered Eating Among Children: A Longitudinal Panel Study. J Commun. 2008;58:361–81 10.1111/j.1460-2466.2008.00389.x

[22] Harrison K, Hefner V Media Exposure, Current and Future Body Ideals, and Disordered Eating Among Preadolescent Girls: A Longitudinal Panel Study. J Youth Adolesc. 2006;35:153–63 10.1007/s10964-005-9008-3

[23] Gallagher D, Visser M, Sepúlveda D, Pierson RN, Harris T, Heymsfield SB How useful is body mass index for comparison of body fatness across age, sex, and ethnic groups? Am J Epidemiol. 1996;143(3):228–39 10.1093/oxfordjournals.aje.a008733 8561156

[24] Flegal KM, Shepherd JA, Looker AC, Graubard BI, Borrud LG, Ogden CL, Comparisons of percentage body fat, body mass index, waist circumference, and waist-stature ratio in adults. Am J Clin Nutr. 2009;89(2):500–8 10.3945/ajcn.2008.26847 19116329 PMC2647766

